# Deep Adaptive Log-Demons: Diffeomorphic Image Registration with Very Large Deformations

**DOI:** 10.1155/2015/836202

**Published:** 2015-05-18

**Authors:** Liya Zhao, Kebin Jia

**Affiliations:** College of Electronic Information & Control Engineering, Beijing University of Technology, Beijing 100124, China

## Abstract

This paper proposes a new framework for capturing large and complex deformation in image registration. Traditionally, this challenging problem relies firstly on a preregistration, usually an affine matrix containing rotation, scale, and translation and afterwards on a nonrigid transformation. According to preregistration, the directly calculated affine matrix, which is obtained by limited pixel information, may misregistrate when large biases exist, thus misleading following registration subversively. To address this problem, for two-dimensional (2D) images, the two-layer deep adaptive registration framework proposed in this paper firstly accurately classifies the rotation parameter through multilayer convolutional neural networks (CNNs) and then identifies scale and translation parameters separately. For three-dimensional (3D) images, affine matrix is located through feature correspondences by a triplanar 2D CNNs. Then deformation removal is done iteratively through preregistration and demons registration. By comparison with the state-of-the-art registration framework, our method gains more accurate registration results on both synthetic and real datasets. Besides, principal component analysis (PCA) is combined with correlation like Pearson and Spearman to form new similarity standards in 2D and 3D registration. Experiment results also show faster convergence speed.

## 1. Introduction

The aim of image registration is to establish spatial correspondences between two or more images of the same/or different scene acquired at different times, from different viewpoints, and/or by different sensors. Usually the ability to capture complex and large image deformations is vital to many computer vision applications including image registration and atlas construction. The problem becomes more challenging when the object in the image or edge of the image undergoes severe deformation [1].

Take medical image registration for example, tissues and organs or body itself are prone to deform, move, and rotate under most circumstances. Most methods iteratively reach a satisfying overlap under specific mathematical criterions, maximizing or minimizing deformation energy as described in 1. Fixed image is defined as *F*, while moving image as *M*. Registration aims to find the optimal model *T* that best satisfies energy *S*. As a result, model *T*, objective function *S* (similarity metric), and optimization method constitutes the three main components of image registration. Consider

(1) $$\begin{array}{c} S\left(F,M\circ T\right)+R\left(W\right). \end{array}$$

According to a state-of-the-art survey [2], registration can be classified into rigid and nonrigid registration. Rigid models restrain the optimum to a few parameters to achieve global registration, while nonrigid models recover local deformation through physical model like elastic or viscous, or statistical model or support vector regression framework, and so forth. In order to fully overlap two images, researchers commonly adopt the two-step strategy, which contains initial registration and following iterative registration [3].

In the two-step strategy, registration firstly begins with a global affine transformation for initial global alignment, take state-of-the-art method FLIRT [4] and ELASTIX [5, 6] for example. Or fiducial markers are firstly detected through feature descriptors, for example, the SIFT method [7], so the initial registration is carried out to establish correspondences between these point sets. In preregistration procedure, rotation, scale and translation of the moving image are modified by the calculated affine matrix. After that, nonrigid registration iteratively goes on. One severe problem of preregistration in affine matrix is that when large distortion and rotation both exists, accuracy is limited by correspondences between those region-based descriptors. If descriptor itself is not accurate, problem becomes more severe. Once descriptors fail to discover point correspondences, accuracy of following registration would be badly influenced. As a result, imprecision may also be introduced misleading the following procedure. Besides, traditional FLIRT and ELASTIX method declares that images for registration must be with the best quality, otherwise poor registration may occur.

**Figure 1:**
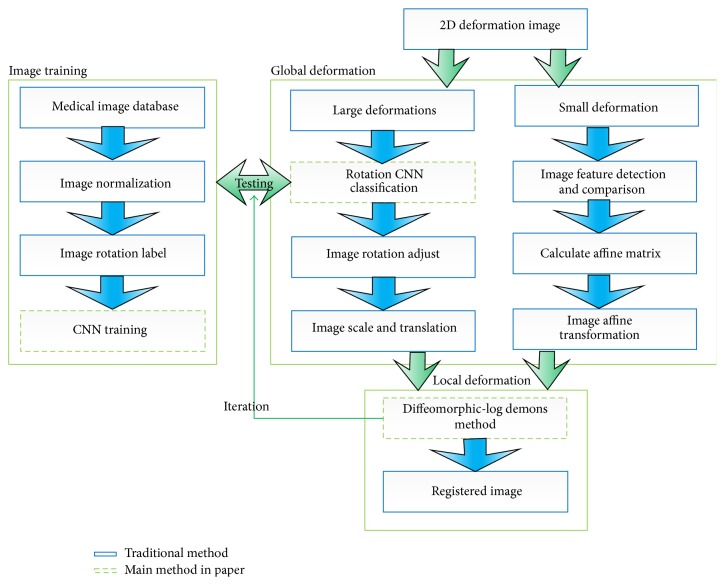
The work flow of 2D image registration.

**Figure 2:**
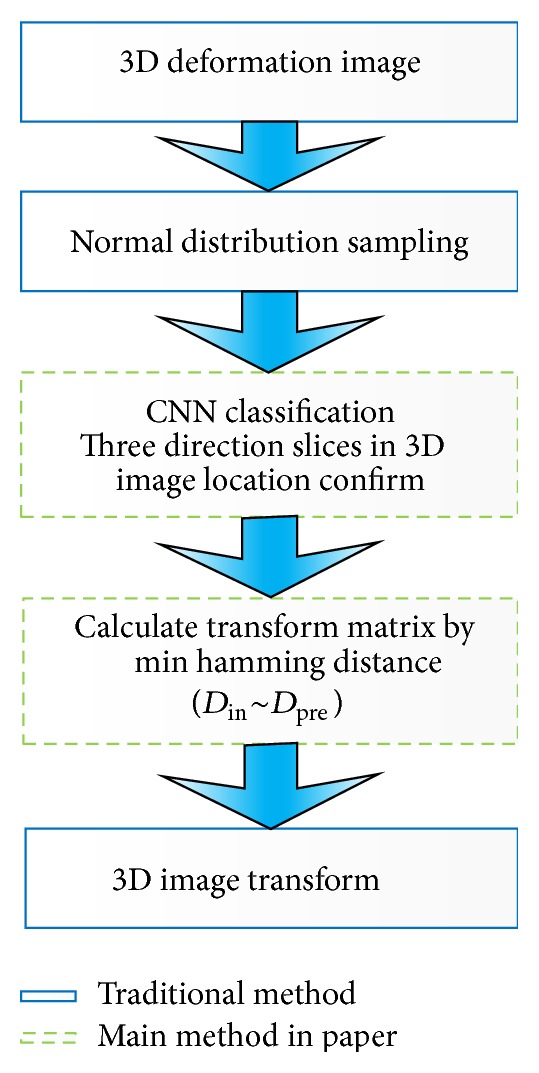
3D image preregistration structure.

In order to address these above limitations and capture very complex and large deformations, we proposed a new approach for image registration based on a two-layer deep adaptive registration framework. Firstly, in the preregistration procedure, rotation, scale and translation extent between two images are obtained separately to achieve initial registration. This is quite different from traditional “one time calculated” affine matrix. For rotation parameter, a CNN classifier is trained offline in order to identify the level of current image rotation under sever distortion. Then scale and translation parameters are obtained. An optimum preregistration is calculated relating to above gained parameters. As for 3D images, a triplanar 2D CNNs [8] around each voxel is utilized for calculation of final affine matrix. Until now, preregistration is done. Secondly, the rectified images are further recovered through the following nonrigid demons registration procedure. In the next circle, the former registration further facilitates results of the later registration of last iteration. This iterative procedure is carried out until an optimum overlap between the two images is achieved. Besides, PCA is introduced to extract the most valuable features, and detected features are put into SSD, Kendall, Pearson and Spearman, and so forth to form new similarity metric. Also, a triplanar 2D PCA is proposed to process 3D registration problem and Figure 1 gives details of the algorithm. As a result, convergence speed is accelerated while maintaining the same registration accuracy. Figures 1, 2 and Algorithm 3 illustrate work flow of our framework of processing 2D and 3D image registration.

The work introduced in this paper contributes in the following aspects:

- Preregistration is improved through estimation of rotation, scale, and translation separately. A multisource CNNs is developed to precisely classify various levels of rotation under sever distortion and help identify rotation extent with high accuracy. For 3D images, triplanar 2D CNNs is constructed to estimate parameters of affine matrix. This new preregistration performs better than state-of-the-art ELASTIX and SURF-based methods.
- A two-layer adaptive registration framework is constructed and it performers better than other so-called two-step strategies.
- PCA is used to extract valuable features and introduced into traditional similarity metric as SSD, Pearson, and so forth. For 3D images, triplanar 2D PCA is proposed to process 3D registration problem. Experiment results show that convergence speed is accelerated with the new similarity standard.
- The proposed framework is tested under both synthetic and nature 2D and 3D images under various extent deformation. Experiment results show that our two-layer deep adaptive registration framework is able to identify the extent of rotation under sever deformation more precisely and correct large and complex distortions with high dice ratio than the comparative methods as it adaptively modify differences between images while others does not have any deep insight of deformation between images.

The rest of the paper is organized as follows. The whole architecture of the proposed two-layer adaptive registration framework for 2D and 3D images is illustrated in Section 2; Section 3 explains methodology of our CNNs classifier preregistration; Section 4 introduces our preregistration in combination with demons nonrigid registration and our new PCA related similarity metric; the proposed methods are evaluated in Section 5 under different datasets and evaluation principles; finally, the conclusion of this work is given in Section 6.

## 2. Architecture

### 2.1. CNNs for 2D Images

The whole workflow of our 2D image preregistration compared with traditional method is illustrated in Figure 1. In traditional algorithms, an affine matrix is calculated through correspondences between detected features, containing information of rotation, scale, and translation. This procedure is significantly influenced by accuracy of detected feature points. And under sever deformation images, traditional feature methods usually corrupt. Our algorithm processes each of the three above elements separately. By refining each procedure, accurate correspondences between fixed and moving image is obtained. It works as follows.

- For rotation, firstly the CNNs classifier is trained offline in order to rectify rotation extent of image under sever deformation. The trained CNNs classifier can identify as much as 360 classes of rotation.
- For scale, image size information is utilized to achieve consistency between fixed and moving image.
- For translation, centroid of each image is calculated through statistical algorithm and translation is achieved by utilizing position information of centroids.

### 2.2. Triplanar CNNs for 3D Images

Different from the 2D image preregistration, CNNs classifier here is used for the slice location of one voxel (*xy*, *yz*, *xz* three directions) instead of the rotation identifier. The work flow of 3D image preregistration is showed in Figure 2. The main procedure includes sampling, slices classification, transform matrix calculation, and image transformation by the matrix. Using CNNs on 3D image registration is a new attempt to resolve image registration for high deformation. Detailed method is introduced in Section 5.2.

## 3. Preregistration

Our strategy consists of firstly preregistration through CNNs classifier on both 2D and 3D images and then utilizing CNNs and demons algorithm adaptively in the following nonrigid registration and finally improving similarity metric for acceleration of registration convergence speed. In this section, we show our preregistration methodology by introducing our CNNs rotation classifier.

### 3.1. Why We Use CNNs

*(1) The Robustness of Classification*. CNNs are a kind of data based classification method which undertakes training by appropriate amount of data. CNN is suitable for nearly any types of data and can make classification with high accuracy, especially for the low quality of fMRI, CT images or images under high deformation (Experiment in Section 5 shows these two kinds of real data are suitable for CNN processing method). Detailed CNNs structure and back propagation training method will be described in Section 3.5.

*(2) Automatic Image Feature Perception*. Nearly all kinds of preregistration method are based on precise feature perception so that the different feature perception methods are playing the key role in this procedure. Traditional image feature perception method is usually based on expert designated data feature. Usually experts give some fixed method to detect specific features of limited kinds of images. For example FLIRT method using inter-model voxel similarity measures where correlation ratio and mutual information are used to detect voxel relationships of different parts. This method has high limitation to the image sources, quality and variable settings. When exceptional case happens, some large deformation images are input for example, it will not work well. While the features from CNNs method are learned by network itself from training data such as edge, brightness, high or low frequency feature, distribution features and so on. Once the training data is updated, the network will get fit for more features automatically at the same time. Although long training time and complicated network variable learning makes CNN method not so easy to use, because of its high accuracy, it is still the image processing trend and future.

*(3) High Efficiency Classification*. Although the data training time of CNNs is long (depending on the detailed training method, network layer structure and hardware equipment like GPU), the total time spent on testing or classification is very short. Once the network is trained well, the only time consuming for processing is as short as linear operation.

Above all, even though there are some good affine transformation methods based on expert knowledge, we still need a smarter one to adapt to more complex image processing tasks in the future.

### 3.2. Theory of CNNs

The concept of deep learning was raised by Hinton and Salakhutdinov [9] in 2006, and it has brought great advances to machine learning since then. Deep learning aims to construct/use brain simulations to recognize data such as image/video, audio and text in an unsupervised way. Deep learning framework uses a multilayer “encoder” network to transform the high-dimensional data into a low-dimensional code and a similar “decoder” network to recover the data from the code. Outputs of low layer network acts as inputs of higher layer network. The whole network aims to equal inputs and outputs without loss of information. By using lower layer features to represent higher layer feature/classification, distributed feature representation of data is found. Auto encoder, Sparse coding, Restricted Boltzmann Machine (RBM), Deep Belief Networks (DBNs) and CNNs are five kinds of deep learning framework. Convolutional neural networks are excellent deep learning architectures, which were firstly introduced by Fukushima [10] and applied for handwritten digit recognition. Image recognition and segmentation tasks have also successfully used CNNs since then, with an error rate as low as 0.23 percent on the MINST database [11]. Besides, it is of high speed and accuracy for image classification in [12]. In facial recognition [13, 14] and video quality analysis [15], CNNs also gained large decrease in error rate and root mean square error.

A CNN is a multilayer perceptron consisting of multilayers, each layer with a convolutional layer followed by a subsampling layer. Through locally connected networks, stationary features of natural images are exploited by the network topology. Firstly, images are sampled into small patches. In the convolutional layers, small feature detectors are learned based on these extracted samples. Then, a feature is calculated by convolution of the feature detector and the image at that point. In the sampling layer, the number of features is reduced to reduce computational complexity and introduce invariance properties. One significant property of features learnt by CNNs is invariance to translation, rotation, scale and other deformations. This twice feature extraction structure enables CNNs with high distortion tolerance when identifying input samples.

### 3.3. CNNs Methodology

The goal of CNNs has no difference with other classification methods. They both focus on minimal total square error. Here we use *c* to denote the class number, and *N* to denote the training dataset, the total square error function can be shown:

(2) $$\begin{array}{c} E^{N}=\frac{1}{2}\overset{N}{\underset{n=1}{\sum }}\;\overset{c}{\underset{k=1}{\sum }}\left(t_{k}^{n}-y_{k}^{n}\right). \end{array}$$

Here *t*_*k*_^*n*^ is the *k*-dimension of the *n* dataset, the *y*_*k*_^*n*^ stands for the *k* output from the network, activation function in CNNs is sigmoid function for faster convergence rate. For each single dataset *n*, 2 can be describes as 3. The final aim of CNNs is to achieve smallest total square error between *t*_*k*_^*n*^ and *y* ^*n*^. Consider

(3) $$\begin{array}{c} E^{n}=\frac{1}{2}\overset{c}{\underset{k=1}{\sum }}{\left(t_{k}^{n}-y_{k}^{n}\right)}^{2}=\frac{1}{2}{\left\|t^{n}-y^{n}\right\|}_{2}^{2}. \end{array}$$

For traditional full connection neural network, BP (Back propagation method) is used to calculate partial derivative to get the minimum square error, usually *I* the current layer, the output of *I* can be shows as 4, where *f* is sigmoid function. Consider

(4)  xl=ful,   with  ul=Wlxl−1+bl,

(5)  xjl=f∑i∈Mjxil−1∗kijl+bjl.

Unlike 4, as 5 shows, for the convolutional layer *I*, the image features (*x*) from prior layer is convoluted by kernel which is different in different layers, *b*_*j*_^*l*^ is the offset of sigmoid function *f*. Consider

(6) $$\begin{array}{c} x_{j}^{l}=f\left(\beta_{j}^{l}\text{down}\left(x_{j}^{l-1}\right)+b_{j}^{l}\right). \end{array}$$

For the sample layer, the image feature numbers and styles are the same with prior layer except the feature size is scaled down. Each feature contains a multi and addition kind offset. The down sample size in this paper is 2 which means the next layer image size is shrink two times by both weight and height. So through combination of 4 and 5, we can get sample equation 7 in which *α*_*ij*_ stands for the value of no. *j* output with no. *i* input features. By calculating *α*_*ij*_ and training kernels by back propagation method we can finally get the best features from different layers with high classification accuracy. Consider

(7) $$\begin{array}{c} x_{j}^{l}=f\left(\overset{N_{in}}{\underset{i=1}{\sum }}\alpha_{ij}\left(x_{i}^{l-1}*k_{i}^{l}\right)+b_{j}^{l}\right). \end{array}$$

Constraint condition ∑_*i*_ *α*_*ij*_ = 1, and 0 ≤ *α*_*ij*_ ≤ 1.

**Figure 3:**
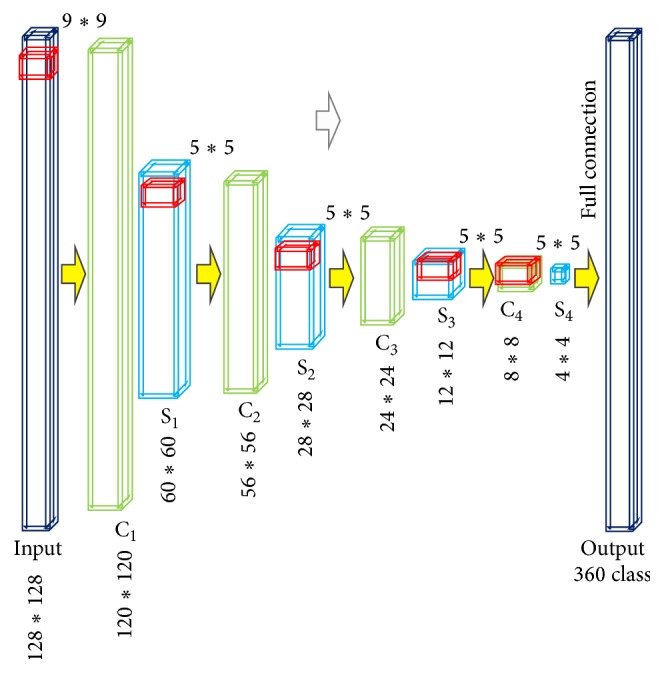
An illustration of CNNs.

As shown in Figure 3, input images are defined as input layer; detailed introduction can be found in Sections 3.4 and 3.5. Hidden layer is the four pairs of convolutional and subsampling layer, which are denoted as *S*_*l*(*l* = 1,2, 3,4)_, *C*_*l*(*l* = 1,2, 3,4)_ and called local connection layer. The output layer is a combination of full connection layer and softmax classifier for classification. Each layer of *S*_*l*(*l* = 1,2, 3,4)_ and *C*_*l*(*l* = 1,2, 3,4)_ is constructed with multi-maps and each map is consisted of multi independent neural cells. Let *d* ^(*l* − 1)^ and *d* ^(*l*)^ be the input and output for the *l*th layer, *S*_*I*_^(*l*)^ × *S*_*I*_^(*l*)^ and *S*_*O*_^(*l*)^ × *S*_*O*_^(*l*)^ be the size of the input and output map, *N*_*I*_^(*l*)^ and *N*_*O*_^(*l*)^ be the number of input and output maps respectively of that layer. According to CNNs, *N*_*I*_^(*l*)^ = *N*_*O*_^(*l* − 1)^ *S*_*I*_^(*l*)^ = *S*_*O*_^(*l* − 1)^.

### 3.4. CNNs Structure Design

We adopt a ten-layer CNNs perceptron network (input and output layers are included; convolutional and sample layers are separately calculated). Key variables setting including kernel size and sample rate of different layers in proposed CNN is showed in Table 1 and Figure 3. Learning rate alpha = 1, variable update batch size = 10, iteration times = 1000, any training and test images are normalized to 128∗128 size gray images with [0,1] pixel size.

### 3.5. Training Image Rotation Classifier through CNNs

Our input images for training are difference images between fixed and moving image: *F* − *M*. *M* is under deformation with different extent of rotation. Each rotation angle of 360° is defined as one class, producing as much as 360 classes. Two distinguishing characters of CNNs are perception field and shared weights. Perception field means each neural cell in each layer is not connected wtih all neural cells in adjacent layers, but limited to a local area of neural cells (9∗9 as in Figure 3). Shared weights means the connection weight parameters (9∗9) of every neural cell to the local area cell are the same. As shown in Figure 3, suppose size of input image *T* is 128∗128. After convolution with filters, the kernel size of which is 9∗9, image changes into Ts1 of 120∗120 size. Image then scales into Tc1 60∗60 in layer S1. After four pairs of *S* and *C*, the original image is represented as Ts4 of only 4∗4 matrix. In this hidden layer, all neural cells on feature maps are not all connected but with same weights. As a result, only 9∗9 weight parameters need to be calculated, greatly reducing computation complexity. An all connection exists between Ts4 matrix and output layer, eliminating disparity caused by partial connection in the hidden layer. Then softmax classifier identifies the matrix and outputs the detected results. After that, the parameters are fine-tuned through back propagation of 1000 times until convergence. After all these steps, a finite classifier is obtained.

## 4. Two-Layer Deep Iterative Registration Framework

### 4.1. Diffeomorphic Log Demons Registration

In the 19th century, Maxwell firstly introduced the concept of demons to illustrate a paradox of thermodynamics. In 1998, Thirion [16] proposed a registration algorithm under demons model, which had a high registration precision and efficiency through pixel velocities caused by edge based forces.

*(i) Theory and Improvements of Demons Registration*. Demons registration utilizes optical flow equation as basis forces for finding tiny deformations in temporal image sequences. For point *p* in space, let *f* and *m* be intensity values in fixed image *F* and moving image *M* respectively. According to Thirion's theory, 8 shows calculation of velocity *u* allowing point *p* to match the corresponding point in *M*. Here, ∇*f* called internal edge force is the gradient image of fixed image and (*m* − *f*) called the external force. In order to make the equation more stable and appropriate for image registration, Thirion added term (*m* − *f*)^2^. Later on, He Wang et al. added image forces of the moving image in the equation to improve convergence speed and stability of the registration as shown in 9. Parameter *α* was proposed by Cachier et al. to adjust force strength. Consider

(8)  u=m−fΔfΔf2+m−f2,

(9)  u=m−fΔfΔf2+α2m−f2+m−fΔmΔm2+α2m−f2.

Vercauteren et al. [17] proposed nonparametric diffeomorphic demons algorithm. It considers the demons algorithm as a procedure of optimization on the whole space of velocity fields and adapts that procedure in a space of diffeomorphic transformations. The transformation result is smoother and more accurate. Then Vercauteren et al. [18] brings the process into log-domain, that is, he uses a stationary velocity field. Besides, the algorithm is symmetric with respect to the order of the input images. Lorenzi et al. [19] implements a symmetric local correlation coefficient to log-demons diffeomorphic algorithm. Lombaert et al. [1] proposed spectral log-demons to capture large deformations. Peyrat et al.  [20] implements multichannel demons to register 4D time-series cardiac images.

*(ii) Diffeomorphic Log Demons Algorithm*. Here, diffeomorphic log demons algorithm is briefly reminded. A diffeomorphic transformation *ϕ* is related to the exponential map of the velocity field *v* : *ϕ* = exp⁡⁡(*v*) (Algorithm 1) [1]. The log-demons framework alternates between optimization of a similarity metric updated by Euler-Lagrangian function in 10. In general, procedure of diffeomorphic log demons framework is described in Algorithm 2. Consider

(10) $$\begin{array}{c} \text{Sim}\left(F,M\circ \exp\left(v\right)\right)={\left\|I_{F}-I_{M\circ \exp\left(v\right)}\right\|}^{2}. \end{array}$$

### 4.2. New Similarity Metric by Combination of PCA

Mathematically, PCA is defined as an orthogonal linear transformation that transforms the data to a new coordinate system to extract the greatest variance in the data set. As a result, it is able to avoid influences caused by image biases. Traditionally, PCA is used for dimensionality reduction to facilitate classification, visualization, communication, and storage of high-dimensional data. Here, PCA is applied in both 2D and 3D medical and usual images, and the detected feature representations are used as inputs of similarity metric to achieve anatomical correspondence and assist optimization procedure in registration.

There are many classical metric measures, such as SSD, mutual information (MI), cross correlation (CC), pattern intensity and also their corresponding improved edition. In this paper, Pearson, Spearman, kendall, SSD together with extracted features by PCA are utilized as the new similarity metric. Pearson, Spearman and Kendall are concepts in statistics and are frequently used in data mining. Pearson is short for Pearson product-moment correlation coefficient (PPMCC), which was developed to measure the linear correlation between two variables. Spearman's rank correlation coefficient is a nonparametric measure of statistical dependence between two variables. Both of their value is between +1 and −1. Spearman has no requirement on variables, while pearson insists variables meets normal distribution. Our utilization of log demons registration avoids the influence brought by this.

**Figure 4:**
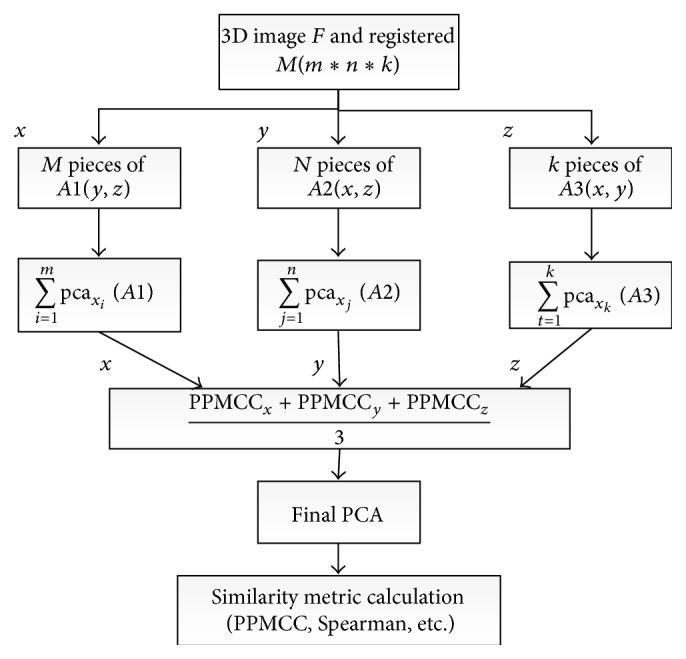
Calculation procedure of 3D PCA-related similarity metric.

- For 2D images of size *m* × *n*, firstly, PCA is applied to both fixed image *F* and registered moving image *M*, gaining pca_*F*_ and pca_*M*_. Thus, most important information of image can be fully utilized by combination of pca_*F*_ and pca_*M*_ as inputs of pearson, spearman, and so forth, forming new similarity metric.
- For 3D images of size *m* × *n* × *k*, firstly, PCA is applied to every slice of *x* axis and gains a series of pca_*x*_*i*__(*i* = 1,2,…, *m*). By summarizing each of  pca_*x*_*i*__(*i* = 1,2,…, *m*),  pca_*x*_ is obtained. The same operation is carried out on *y* and *z* axis data, obtaining pca_*y*_, pca_*z*_. Then, PCA of both fixed (*f*_*x*_, *f*_*y*_, *f*_*z*_) and registered moving (*m*_*x*_, *m*_*y*_, *m*_*z*_) image is calculated. Thus, information of image can be fully utilized by combination of (*f*_*x*_, *f*_*y*_, *f*_*z*_) and (*m*_*x*_, *m*_*y*_, *m*_*z*_) as inputs of PPMCC, Spearman, and so forth. Workflow of this part is shown in Figure 4.

### 4.3. Two-Layer Iterative Registration Framework

Traditionally, the two step registration means an initial affine registration in the very beginning to coarsely rectify deformation and a following iterative registration to optimize a similarity metric achieving fine registration. We also adopt the two step strategy. But before the two step registration, we build up a classifier offline under CNNs training to identify rotation between fixed *F* and moving *M* image under very large distortions, then scale and translation. Also in each iteration, the initial and following registration are carried out iteratively. This feed-back procedure assists achieving higher registration accuracy comparing with traditional SURF and affine method.

Besides, at the end of each iteration, we utilized a new similarity metric by combining PCA with traditional SSD, pearson, and so forth, fully containing most important features of image. As a result, convergence speed is highly accelerated than traditional SSD without PCA while maintaining the same registration accuracy. Algorithm 3 shows the over flow of the framework.

## 5. Experiment Results

In this section, the performance of the whole two-layer registration method is evaluated on both 2D and 3D images, synthetic and nature datasets. For comparison, traditional two step methods, ELASTIX and SURF related algorithm are used to preregistrate moving and fixed image. Then demons nonrigid registration is conducted. These methods are set as the baseline methods, which are denoted as ELASTIX+demons and SURF+demons. They all firstly use detected features initially to register images through affine transformation and original SSD as similarity metric under the diffeomorphic log demons framework. Our method is different from their framework both in preregistration and following nonrigid registration framework. For 2D images, firstly train a rotation classifier through CNNs and preregister moving image under large distortion and rotation, then together with scale and translation transformation, preregistration is done. For 3D images, a pretrained triplanar 2D CNNs is utilized to locate voxels, establishing feature correspondences. Finally, PCA related similarity metric iteratively registering images under diffeomorphic log demons framework.

The improvement of our two-layer method in registration accuracy, robustness to large deformation and rotation, and convergence speed are all assessed with ground truth data. Our matlab code is under Lombaert's work [21] and Toolbox [22].

Specifically, we downloaded brain and lung dataset from BrainWeb MRI Simulated Normal Brain Database [23, 24] and Empire 10 challenge [25] for training and testing. Besides, training and tests were also carried out on classical Lena image, which is mostly used for image processing. T1, T2 image from [26] and ITK image [27] is also utilized in our experiment. BrainWeb MRI dataset contains 20 both 2D and 3D new normal anatomical models. Empire 10 challenge lung dataset includes 20 3D scans, each containing fixed and moving image pair. Description of lung dataset is shown in Table 2 [28].

### 5.1. 2D Image Registration

#### 5.1.1. Synthetic Deformation Tests

A lot of registration has been evaluated on synthetic deformation images for algorithm test according to previous work [1, 26].

**Figure 5:**
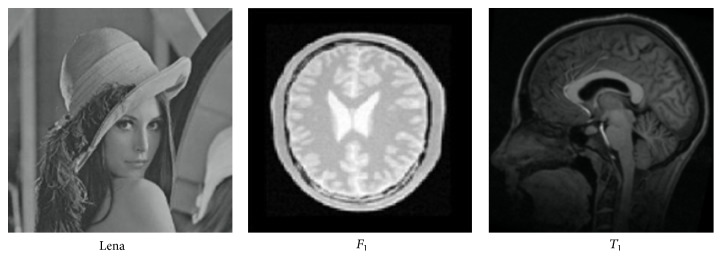
An example of original sample image.

**Figure 6:**
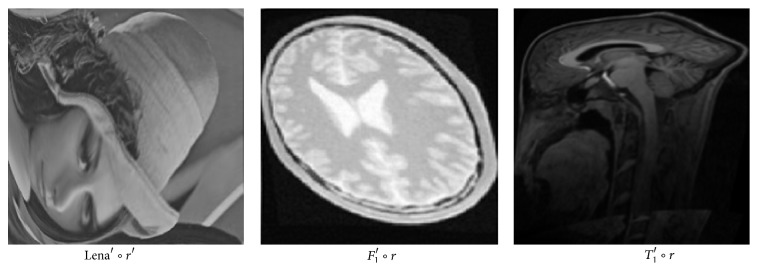
An illustration of sample image after sever distortion and large rotation.

*(1) Training CNNs Deformation Classifier*. In this work, ten-layer CNNs are constructed to train various sources of images. We also tested other number layer CNNs, results showed that ten-layer CNNs achieved highest score when classifying rotation of deformed images. Four kinds of 2D source images [24–27] served as samples. An example of sample image is shown in Figure 5. Take image *T*_1_ for example, linear transformation like rotation or translation is added to image *T*_1_ by multipling rotation matrix *r* coded through matlab; then four kinds of large and complex nonlinear transformation is added to *T*_1_ through special processing by photoshop. *T*_1_ image with only rotation is denoted as *T*_1_∘*r*, with only deformation noted as *T*_1_′, and with both rotation and deformation denoted as *T*_1_′∘*r*. The same notation is with *F*_1_ and Lena image.  Figure 6 is an illustration after all those processing. In order for accurate identification of rotation, here for training, difference image of *F* and *M* (with only rotation) *D*_*T*_1__ = ‖*T*_1_ − *T*_1_∘*r*‖ is input of CNNs. After training, each angle of 360° is defined as one class, obtaining 360 class of distortion. For other CNNs, number of classes is 180, 90, 36.

Our test is carried out on computer of windows 7 system, with 8 GB RAM, i7-4770 CPU @ 3.4 GHz. Take BrainWeb data [23, 24], for example, Table 3 shows test results of the classifier according to these data.

As we can see from Table 3, when input images are resized into 64 × 64 pixels, identification of rotation can reach as much as 99.86% for classifier 90; while images are resized into 28 × 28 pixels, the identification accuracy for classifier 36 is 99.97%. All these are done under condition that training data is also for testing. When the testing data of BrainWeb is put into the trained classifier, accuracy reaches 99.56%, even lower than the training data itself, but still very high according to many usual classifiers. For Lena, ITK and T1 training data, classifier 36 gains an accuracy rate of 99.94%. Number of iteration is set to 1000 for every training.

*(2) CNNs Preregistration Test*. SURF related method, ELASTIX and our CNNs method are tested. Here, SURF related method means using firstly SURF algorithm to detect features and then affine transformation to initially register images.

**Figure 7:**
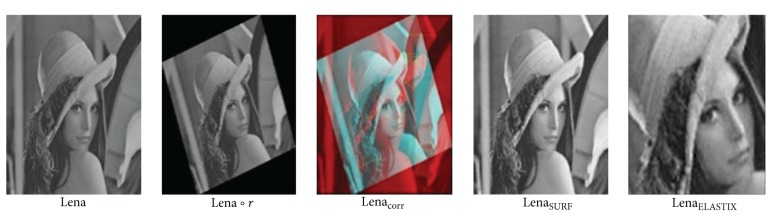
Preregistration result of ELASTIX and SURF method with only rotation on image.

**Figure 8:**
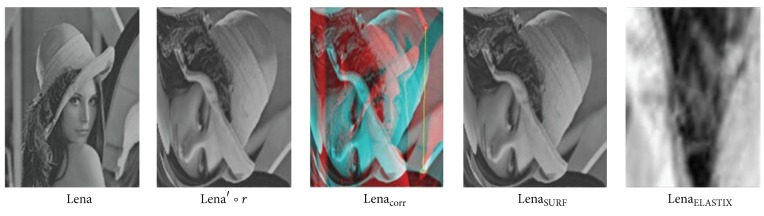
Preregistration result of ELASTIX and SURF method with both rotation and large deformation on image.

**Figure 9:**
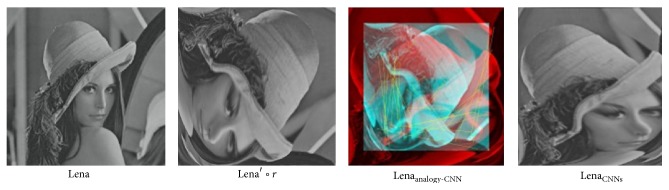
Preregistration result of CNNs method with both rotation and large deformation on image.

- When only rotation exists as Lena∘*r* in Figure 7, ELASTIX method failed; SURF method is able to identify rotation invariant features and establish accurate correspondences between Lena and Lena∘*r*. Established correspondences are shown as Lena_corr_ in Figure 7. Vertices of the yellow lines stand for feature correspondences. Red circle vertex stands for feature in original moving image Lena∘*r* while green cross stands for corresponding feature point in registered moving image Lena_SURF_. Registered image is denoted as Lena_ELASTIX_ and Lena_SURF_.
- However, when rotation and large deformation simultaneously appears in moving image as Lena′∘*r* in Figure 8, both ELASTIX and SURF method crushed. Under such circumstances, in our tests SURF only found one pair of correspondence points. As there are not enough feature correspondences, initial registration failed.
- On the contrary, our trained CNNs classifier and following scale and translation operation directly identified Lena image's rotation angle accurately (90°  rotation), and turned it back to Lena′ as in Figure 9. For better comparison, we used software to show ways of rotation processing in CNNs as SURF's manner, feature detecting and matching in Lena_analogy - CNN_. As enough number of so-called features are detected, CNNs is able to recover rotation added to Lena′.

*(3) Accuracy Evaluation of Registration*. Mathematically, dice ratio is used to evaluate overlap between two datasets. It is defined in 11. In this section, both dice ratio and subjective human evaluation method is used to assess accuracy of ELASTIX and SURF related registration and our method result

(11) $$\begin{array}{c} O_{\text{overlap}}=\frac{2F\cap M}{F+M}. \end{array}$$

After preregistration in Section 5.1.1, ELASTIX and SURF related method performs diffeomorphic log demons algorithm iteratively to achieve for best registration; while our method iteratively carries out CNNs classifier and diffeomorphic log demons algorithm to optimize registration. This new two-layer registration framework makes full use of both preregistration and following demons method and registration results show that it indeed improves accuracy.

**Figure 10:**
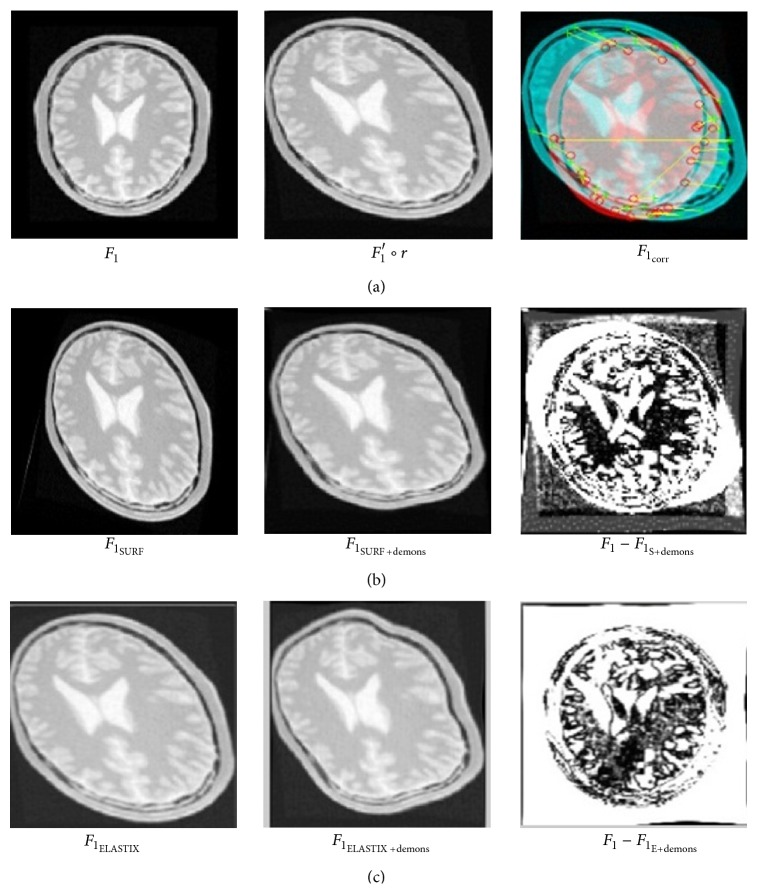
Preregistration result of SURF related method (b) and ELASTIX method (c) with both rotation and large deformation on image.

**Figure 11:**
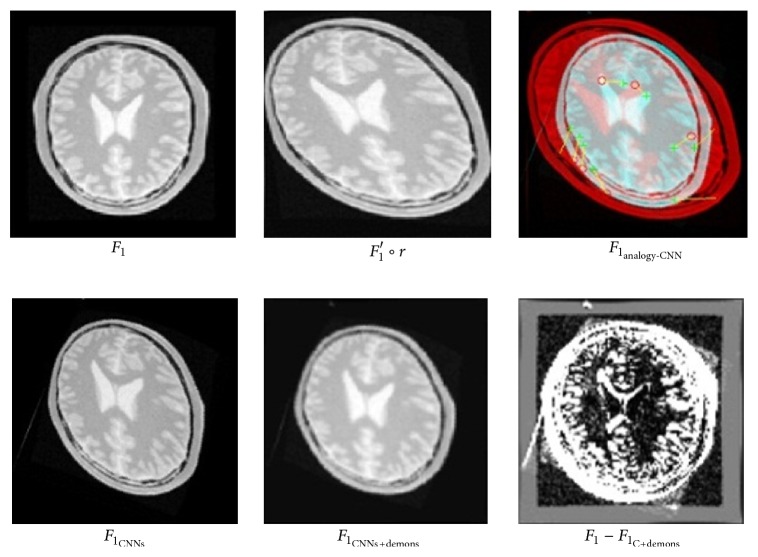
Preregistration result of CNN method with both rotation and large deformation on image.

Figure 10 shows registration procedure and result of ELASTIX (Figure 10(c)) and SURF (Figure 10(b)) related method, while Figure 11 shows that of our method. When both rotation and deformation exists in image *F*_1_, our registration result *F*_1_ − *F*_1__C+demons_ is much better than *F*_1__ELASTIX+demons_ and *F*_1__SURF+demons_ apparently. Besides, to test dice ratio of registration, original fixed image *F*_1_ and registered moving image of two methods are put into function 11 separately. Dice ratio of ELASTIX and SURF-demons method is 0.889 and 0.88, while our CNNs-demons-iterative method achieves as much as 0.8964.

#### 5.1.2. Lung Atlases

**Figure 12:**
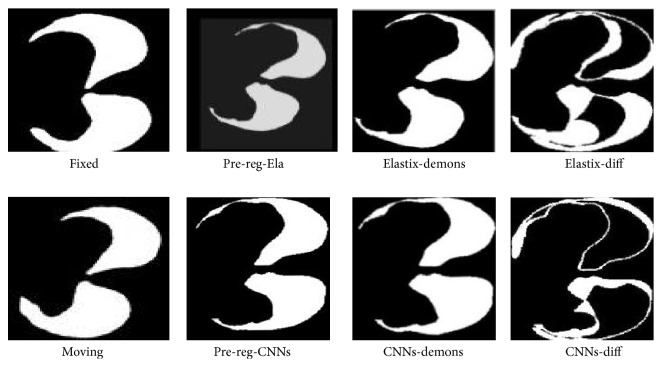
Lung slice 8 registration.

**Figure 13:**
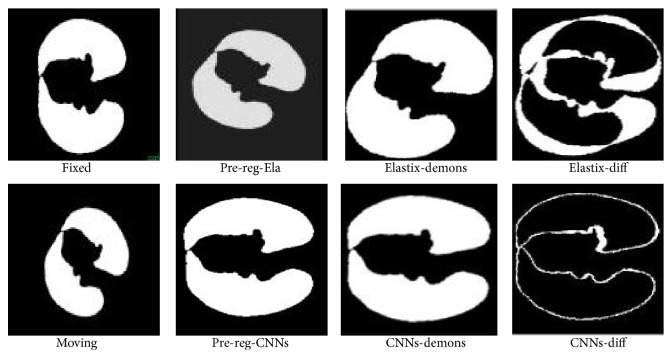
Lung slice 6 registration.

Description of lung dataset can be found in Table 2 [28]. Empire 10 lung datasets are firstly used for the MICCAI conference 2010. It contains 20 intra-patient thoracitic CT image pairs. Figures 12 and 13 shows our preregistration results of slice image 8 and 6 of 4D image pairs compared with that of Elastix tool. All images are shown with the help of tool vv [29]. From left to right in the figures are fixed image, moving image, preregistration result of ELASTIX and our proposed CNNS method, final registration result of above two methods with demons. It is obvious that our method can accurately rectify rotation, scale and translation deformation added to moving image. While ELASTIX preregistration failed to rectify rotation differences between fixed and moving images. Registered images are denoted as Elastix-demons and CNNs-demons. Diff images between fixed and registered image are denoted as Elastix-diff and CNNs-diff.

**Figure 14:**
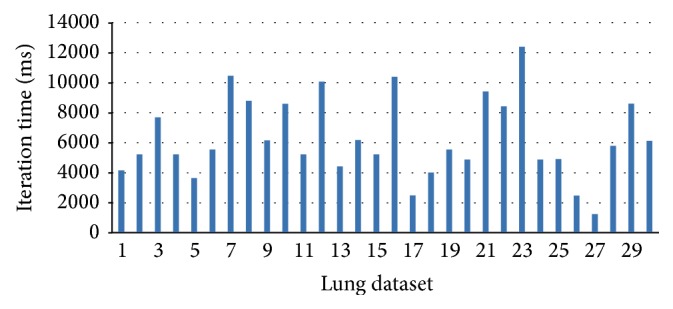
ELASTIX preregistration consuming time of the 30 slices of 4D lung dataset.

ELASTIX preregistration consuming time of each slice is shown in Figure 14. The shortest time of one slice is more than 1000 ms (1 s) and time for slice 8 is 3500 ms. Although training of our CNNs classifier costs long time, it is offline. And our CNNs rotation, scale and translation operation costs a total of only 39 ms. As a result, it is quite attractive for real-time clinical applications.

#### 5.1.3. Brain Atlases

**Figure 15:**
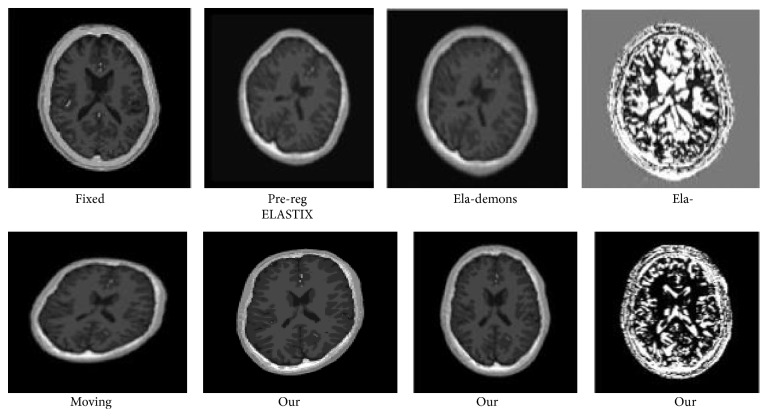
Brain slice registration.

We select the cross section 2D image of the BrainWeb MRI 20 object, 10 for training and the other 10 for testing. From Figure 15, we can see that our proposed preregistration can rectify both rotation and translation more successfully than traditional Elastix affine registration.

### 5.2. An Attempt on 3D Image Registration by Using CNNs

**Figure 16:**
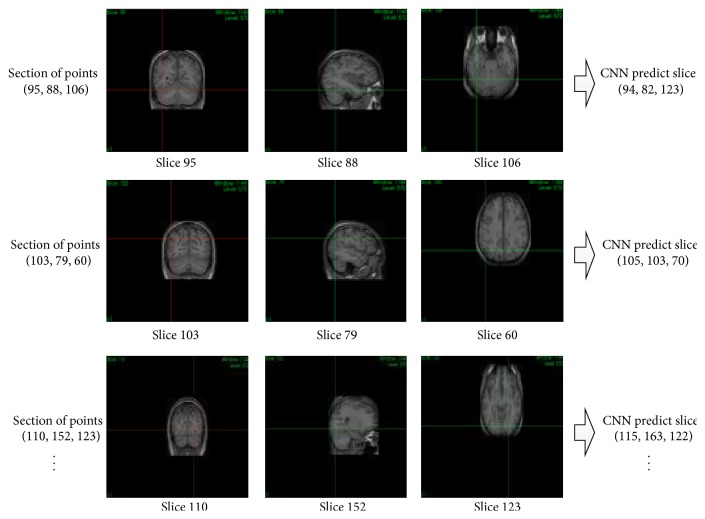
3D Sample voxel slice images (three slices, *xy*, *yz*, *xz*) classification.

For the 3D image registration part we focus on the brain atlases registration and give a CNNs 3D image registration method. We train brain atlas from 18 people's 3D image data in BrainWeb Brain database by four steps: (1) Randomly select 10 label points by Normal distribution in 3D image. (2) Adjust 3D brain image and separate it to 2D image on three directions (*xy*, *yz*, *xz*). (3) Test each 2D slice position by triplanar after-trained CNNs classifier (each dimension enjoys one CNN network) and get the right slice position (predicted voxels). (4) Adjusting the 3D image to make label voxels and predict voxels that enjoy smallest hamming distance. Experiments shows the high accuracy CNNs classify results will greatly improve moving 3D image's similarity to the fixed 3D image. The detailed procedure is shown in Figure 16.

### 5.3. Convergence Speed Evaluation of Registration

**Figure 17:**
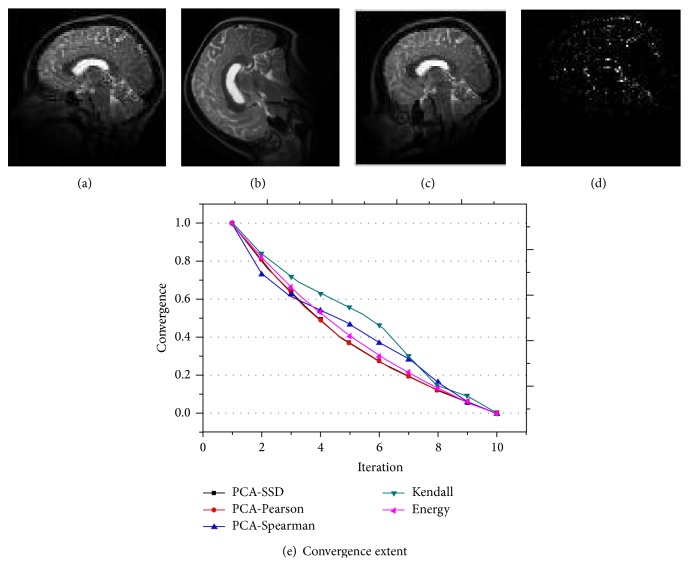
T2 Data: (a) fixed image, (b) moving image, (c) registered moving image, (d) difference between (a) and (c), (e) convergence extent of the first ten iteration.

Sections 5.1 and 5.2 improve both registration accuracy and speed. In this section, we test registration accuracy on T2 brain medical data and focus on accelerating convergence speed of registration. PCA is introduced to extract valuable features and by combining features with SSD, Pearson, Spearman, Kendall, we get new similarity PCA-SSD, PCA-pearson, PCA-spearson and Kendall. Original SSD is denoted as energy in Figure 17(e). The course-to-fine (in here, three level is recommended) registration strategy is adopted in here. In Figure 17, horizontal axis stands for iteration times and vertical axis stands for the values of metric. Firstly, mean convergence extent of the three-level registration is calculated. Then normalization is carried out on the mean value. Several conclusions can be gained:

1. both PCA related and original SSD methods converge regularly,
2. as a whole, PCA-SSD and PCA-Pearson methods perform best and converge faster than original SSD metric;
3. PCA-spearman metric firstly converges fastest, but latterly it slows down;
4. Kendall metric performs worst compared with other metrics.

## 6. Conclusion

In this paper, a comprehensive method of constructing rotation classifier for images under severe deformation and rotation was proposed through CNNs. The classifier is able to identify distortion as much as 360 classes according to analysis of rotation angles. The classifier is utilized to assist our proposed two-layer deep adaptive registration framework. In each registration iteration, preregistration with identification of the trained classifier, scale, and translation operator and following diffeomorphic log demons registration facilitates each other one after another. Besides, proposed PCA related similarity metric helps achieve faster convergence speed. The new two-layer registration framework is compared with traditional diffeomorphic log demons registration in combination with state-of-the-art ELASTIX and SURF preregistration. As baseline method carries out preregistration only once, large deformations cannot be fully modified. From tests on different image resources containing various kinds of both 2D and 3D, MRI, and CT datasets, our framework indeed outperforms the baseline method on both registration quality and convergence speed.

In the following work, we would combine other kinds of deep learning framework as independent subspace analysis (ISA) [30], sparse coding [31], and so forth to improve current registration. Also, more performance tests of the proposed two-layer registration framework should be carried out on more data resources. Besides, the proposed method performance should be compared with other deep learning models.

## Figures and Tables

**Algorithm 1:**
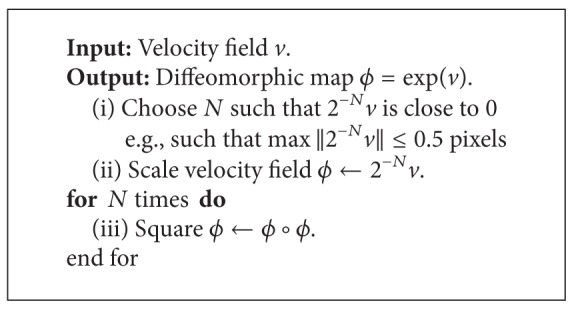
Exponential *ϕ* = exp⁡(*v*) [1].

**Algorithm 2:**
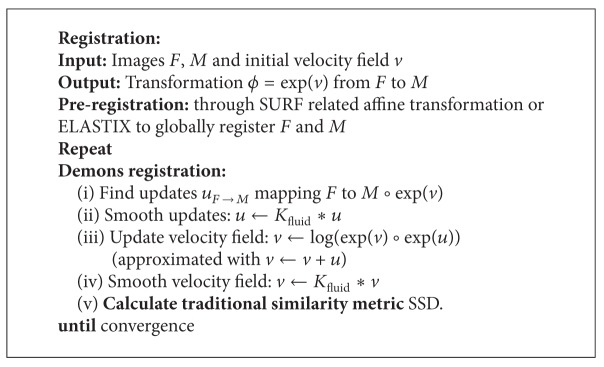
SURF/ELSTIX related registration framework.

**Algorithm 3:**
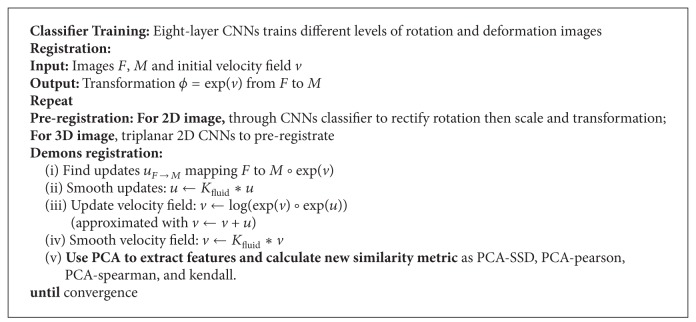
Two-layer unsupervised deep adaptive registration framework.

**Table 1:** Key setting variables in CNN network.

Layer	Name	Kernel size or sample rate
1	Input layer	None
2	1st convolutional layer	9∗9
3	1st sample layer	2
4	2nd convolution layer	5∗5
5	2nd sample layer	2
6	3rd convolutional layer	5∗5
7	3rd sample layer	2
8	4th convolution layer	5∗5
9	4th sample layer	2
10	Output layer	None

**Table 2:** Listing of data used in lung registration [28].

Pair	Data category	Pair	Data category	Pair	Data category
1	Insp-Exp	11	Insp-Insp	21	Insp-Exp
2	Insp-Insp	12	Warped	22	Insp-Insp
3	Insp-Insp	13	4D	23	4D
4	Ovine	14	Insp-Exp	24	Ovine
5	Warped	15	Insp-Insp	25	Warped
6	Contrast	16	4D	26	Contrast
7	Insp-Exp	17	4D	27	Insp-Insp
8	Insp-Exp	18	Insp-Exp	28	Insp-Exp
9	Insp-Insp	19	Insp-Insp	29	Ovine
10	Ovine	20	Insp-Exp	30	Warped

**Table 3:** Performance of classifier.

	Image size	Accuracy of classifier 36	Accuracy of classifier 90	Time (s each iteration)
BrainWeb Training data	64 × 64	*※*	99.86%	41.2
	28 × 28	99.97%	*※*	6.17
BrainWeb Testing	28 × 28	99.56%	*※*	*※*
Lena, ITK, T1	28 × 28	99.94%	*※*	2.4
